# Is the Subthalamic Nucleus Sleeping Under Nitrous Oxide–Ketamine General Anesthesia?

**DOI:** 10.1111/ejn.70039

**Published:** 2025-03-05

**Authors:** Halen Baker Erdman, Hagai Bergman, Karin Abu Haya, Stefanie Glowinsky, Lotem Warhaftig, Juan F. León, Zvi Israel, Muneer Abu Snineh, Evgeniya Kornilov, Omer Zarchi, Idit Tamir, Johnathan Reiner, Tsvia Fay‐Karmon, Sharon Hassin‐Baer, Violeta Glauber, Tomer Nir, José Antonio Asprilla González, Lior Ungar, Zion Zibly

**Affiliations:** ^1^ Department of Medical Neurobiology Hebrew University of Jerusalem, Ein Kerem Campus Jerusalem Israel; ^2^ Edmond and Lily Safra Center for Brain Sciences Hebrew University of Jerusalem, Givat Ram Campus Jerusalem Israel; ^3^ Department of Neurosurgery Hadassah Medical Center Jerusalem Israel; ^4^ Surgical Monitoring Services Bet Shemesh Israel; ^5^ Department of Neurology Hadassah Medical Center Jerusalem Israel; ^6^ Department of Anesthesiology Rabin Medical Center Petach Tikvah Israel; ^7^ Department of Neurobiology Weizmann Institute of Science Rehovot Israel; ^8^ Department of Neurosurgery Rabin Medical Center Petach Tikvah Israel; ^9^ Department of Neurology Rabin Medical Center Petach Tikvah Israel; ^10^ Movement Disorders Institute and Department of Neurology Sheba Medical Center Ramat Gan Israel; ^11^ Department of Anesthesiology Sheba Medical Center Ramat Gan Israel; ^12^ Department of Neurosurgery Sheba Medical Center Ramat Gan Israel; ^13^ Department of Neurosurgery Yale Medical Center New Haven Connecticut USA

**Keywords:** deep brain stimulation, electrophysiology, ketamine sedation, nitrous oxide, subthalamic nucleus

## Abstract

Nitrous oxide is a common gaseous anesthetic used in a wide range of medical procedures due to its desirable combination of anesthetic and analgesic properties. Deep brain stimulation surgery, a well‐established treatment for movement disorders like Parkinson's disease, often requires precise microelectrode recordings of the awake brain's electrical signals for optimal results. However, the influence of anesthetics on these brain signals remains a critical consideration. This study investigated how nitrous oxide general anesthesia supplemented by ketamine affects the electrophysiology of the subthalamic nucleus compared to awake and low‐dose ketamine sedation during deep brain stimulation procedures targeting the subthalamic nucleus of Parkinson's disease patients. Spectral analysis of subthalamic nucleus electrophysiological characteristics and statistical analysis of its electrophysiological dimensions were performed on retrospective data from three medical centers. Our findings revealed that nitrous‐ketamine general anesthesia allows electrophysiological subthalamic nucleus identification, despite a slight decrease in overall activity level. Nevertheless, nitrous–ketamine showed significantly lower beta frequency power inside the nucleus compared to the ketamine and awake groups. At the group level, and in many trajectories, delineation of subthalamic nucleus subdomains can be achieved by detection of changes in the delta frequency oscillations. Notably, no differences in electrophysiological nucleus dimensions were found between the three groups. These findings suggest that it is possible to recognize the entrance and exit of the subthalamic nucleus with high confidence under nitrous oxide–ketamine anesthesia. However, the motor subregion of the nucleus is more difficult to delineate under nitrous anesthesia than ketamine sedation or awake, which may affect outcome.

## Graphical abstract

Nitrous oxide–ketamine general anesthesia allows subthalamic nucleus (STN) border detection but disrupts classical STN motor/non‐motor subregion delineation in Parkinson's disease patients undergoing deep brain stimulation (DBS) surgery. Firing rate is decreased, but firing pattern is preserved. Given these results and the desire to improve patient and surgeon experience, the use of N_2_O–ketamine anesthesia in DBS procedures is valuable for selected cases.

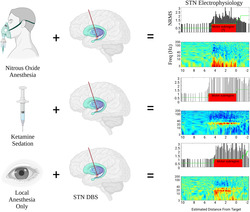

**Abbreviations**
ANOVA: analysis of variance
AUC: area under the curve
CV‐ISI: coefficient of variation of interspike interval
DBS: deep brain stimulation
ENIGMA: Evaluation of Nitrous Oxide in the Gas Mixture for Anesthesia
ERNA: evoke resonant neural activity
GPi: globus pallidus internal segment
MER: microelectrode recording
N_2_O: nitrous oxide
NRM: normalized root mean square
PD: Parkinson's disease
PSD: power spectrum density
SEM: standard error mean
STA: spike‐triggered average
STN: subthalamic nucleus
TIVA: total intravenous anesthesia

## 1 Introduction

Nitrous oxide (N_2_O) is one of the oldest known inhaled anesthetics. Today, it is commonly used in various clinical settings due to its combination of anesthetic and analgesic properties. N_2_O also has a low solubility in blood and tissue, leading to fast induction and recovery. It is not a potent drug and, in fact, needs 104% concentration to reach a minimum alveolar concentration of 1 in healthy adult patients (Mapleson 1996). For this reason, it is frequently given in conjunction with other anesthetics when deeper anesthesia is needed. At low doses, N_2_O leads to a sedative dissociative‐like state. The mechanism of action of N_2_O's anesthetic effects is multifaceted but, like ketamine, is known to be a noncompetitive NMDA receptor antagonist, suggesting a dissociative mechanism.

In line with the NMDA antagonism dissociate effects, EEG studies (Foster and Liley 2013) revealed that inhalation of 40% N_2_O causes a total power decrease in frontal sites and particularly attenuates the delta frequency band. This finding is unique from other common anesthetics which lead to an increase in delta frequency power in frontal regions. Increases of 40–50 Hz (narrow gamma range) power have also been reported as a result of N_2_O anesthesia (Rampil et al. 1998). A rebound effect of increased frontal theta frequency is then observed after administration of the drug is stopped (Foster and Liley 2013). Similar gamma frequency increases are reported for ketamine anesthesia (Akeju et al. 2016) and sedation (Guang et al. 2021; Slovik et al. 2017).

The safety of N_2_O has been evaluated in various studies, the largest of which has been the Evaluation of Nitrous Oxide in the Gas Mixture for Anesthesia (ENIGMA‐I, ENIGMA‐II; Myles et al. 2007, 2009, 2014) trials. However, the results are incongruent for many of the measurements across ENIGMA‐I and ENIGMA‐II. ENIGMA‐I showed an increase in many complications following N_2_O anesthesia including myocardial infarction, stroke, pneumonia, pulmonary embolism, wound infection, and death, but ENIGMA‐II found no increases. There does not seem to be an effect on long‐term neurological outcomes, even following neurosurgery (Ko et al. 2014). Despite the lack of consensus about its potential complications, N_2_O remains a widely used anesthetic across many settings.

Deep brain stimulation (DBS) is a surgical procedure involving the placement of a stimulating brain lead for the treatment of movement disorders, epilepsy, and psychiatric disorders. Currently, the majority of DBS surgeries are performed on Parkinson's disease (PD) patients and generally target the subthalamic nucleus (STN) or globus pallidus internal segment (GPi). PD patients are usually recommended for DBS when optimal therapy fails to provide a satisfactory quality of life or after the side effects of dopamine replacement therapy become intrusive. Results of the surgery are dramatic decreases in the motor symptoms of PD including tremor, bradykinesia, and rigidity. These results have been shown to persist for more than 15 years following DBS surgery (Merola et al. 2014).

Whereas severe adverse effects from DBS surgery are rare, moderate adverse effects such as dysarthria are more common (Limousin and Foltynie 2019). These moderate adverse effects could result from sub‐optimal lead placement within the target nucleus. Indeed, retrospective studies of two big clinical databases revealed a significant fraction of correction procedures due to lead mislocalization (Okun et al. 2005; Rolston et al. 2016). One way to improve targeting is to use microelectrode recording (MER) to establish the precise location of the nucleus in the operating room rather than relying on pre‐ or intraoperative imaging, which are more limited in their spatial resolution and, in the case of preoperative imaging, can be affected by brain shift (Halpern et al. 2007; Oxenford et al. 2022). However, MER is most reliably performed with the patient awake, rather than anesthetized (Benady et al. 2020; Krishna et al. 2015; Raz et al. 2010; Sheshadri et al. 2017), so as not to cause changes in the electrophysiology of the target structures (Bergman et al. 1994; Deffains et al. 2016). The awake patient also allows for intraoperative stimulation testing, so a good therapeutic window is ensured, and adverse effects can be avoided. This idea was challenged recently by the finding that conscious sedation using low‐dose ketamine during MER in DBS surgeries does not significantly disturb the electrophysiological signature of the STN and permits the patient to undergo intraoperative stimulation testing (Baker Erdman et al. 2022; Kornilov et al. 2024). Yet, some patients require general anesthesia due to systemic health issues, their neurological status, age, and personal preferences. Here, we study how N_2_O–ketamine general anesthesia affects the electrophysiology of the STN in PD patients compared to awake and low‐dose ketamine sedation.

## 2 Material and Methods

### 2.1 Participants

Data were collected from 112 patients across three Israeli medical centers, totaling 192 MER trajectories. All patients were diagnosed with idiopathic PD and were selected for DBS surgery targeting the STN. Patient demographics and preoperative status are shown in Table 1. In some cases, demographics or preoperative status could not be found and were omitted from this analysis.

**TABLE 1:** Patient demographics and preoperative status.

	Awake (Hadassah Medical Center)	Ketamine (Rabin Medical Center)	N_2_O (Tel Hashomer Medical Center)
Patient demographics			
Number of patients	35	40	37
Age, years	64.6 (10.5)	66.7 (9.0)	66.9 (10.0)
Sex, M:F	23:12	27:13	29:8
Preoperative status			
Disease duration, years	10 (5.9)	7.6 (3.6)	8.9 (4.3)
Daily levodopa equivalent, mg^a^	1020 (518.1)	1026 (524.5)	803.3 (384.8)
UPDRS‐III off medication	40.5 (8.6)	43.4 (16.6)	44.5 (12.3)
UPDRS‐III on medication	20.1 (8.6)	21.9 (11.7)	33.3 (13.9)

*Note:* STD shown in parentheses.

Abbreviation: UPDRS‐III = Unified Parkinson's Disease Rating Scale part III score.

^a^ Denotes significant difference.

### 2.2 Study Design

This retrospective study aimed to investigate the electrophysiological alterations that occur in the STN of PD patients undergoing DBS surgery with N_2_O anesthesia with supplemental low‐dose ketamine compared to only low‐dose ketamine sedation and awake patients. Patients were divided into three groups for the purposes of this study based on the anesthetic protocol they underwent during the MER stage of their DBS surgery. The awake group consisted of 35 patients (62 trajectories) operated at Hadassah Medical Center. The ketamine group consisted of 40 patients (67 trajectories) operated at Rabin Medical Center. These patients were given low‐dose ketamine sedation during MER recording and low‐dose propofol sedation before/after the MER period. The N_2_O group consisted of 37 patients (63 trajectories) who underwent DBS surgery at Sheba (Tel Hashomer) Medical Center. These patients were given N_2_O general anesthesia (and if needed ketamine augmentation at doses similar to those given in the ketamine group) before and during MER.

### 2.3 Ethics Statement

The study was approved by the hospitals' Helsinki committees (ethics numbers 0569‐20‐RMC and 0328‐20‐RMC: Rabin Medical Center, 0168‐10‐HMO: Hadassah Medical Center, 9283‐22‐SMC: Sheba (Tel Hashomer) Medical Center). Patient consent was waived due to the retrospective nature of this study.

### 2.4 Surgical Protocol

The surgical protocol is described in full in Baker Erdman et al. (2022). Briefly, the STN was targeted by the surgeons using a preoperative 3‐T T2 MRI. The axial plane with the largest diameter of the red nucleus was selected, and the STN target was set on the medial to central STN and along Bejjani's line in this plane. Trajectory default angles were 60° and 20° from the axial and midsagittal planes and adjusted to cortical, ventricle, and blood vessel anatomy (using T1 protocol with gadolinium). Patients stopped taking medications either the evening before in the case of a morning surgery or the morning of in the case of an afternoon surgery. On the day of surgery, a stereotactic frame (CRW or Leksell) was attached to the patient's head, and a high‐resolution CT scan was performed. The CT images were then coregistered with the preoperative MRI scans, and the stereotactic frame coordinates and angles were extracted using Medtronic Stealth 8 or Brainlab iPlanNet Version 3. Patients were then transferred to the operating room and positioned supine, and their heads were fixed to the operating table by the stereotactic frame.

Though exact surgical protocol varied across centers, generally, the patient was given a subcutaneous injection of local anesthetics at the planned incision site, and a skin incision was made, followed by a burr‐hole at the planned entrance point using the stereotactic frame coordinates. In some centers, these steps were performed for both hemispheres before continuing, but in others, the procedure was performed for each hemisphere separately. The dura, arachnoid, and pia were then cut and coagulated, and 1–2 guide tubes were lowered to 15 mm above the planned target. Recording microelectrodes (NeuroProbe, Alpha Omega Engineering, Nof HaGalil, Israel; impedance at 1000 Hz = 300–900 Kohms) were lowered through the guide tubes extending 5 mm past the end of the guide tube. MER navigation to the STN was then performed by an expert electrophysiologist that was consistent across all centers (H.B.E.). The recording electrode was then replaced by a permanent DBS lead (either 3389 or SenSight, Medtronic, Minneapolis, MN, or Vercise, Boston Scientific, Marlborough, MA). Leads were secured to their skull fixation device; the incisions were closed, and the stereotactic frame was removed from the patient's head. Tunneling and implantation of the internal pulse generator were usually carried out on the same day.

### 2.5 Anesthetic Protocol

All patients were given local anesthesia infiltration (lidocaine 2% and bupivacaine 0.5%, ~20 cc) and mild sedation (2 mg IV midazolam) prior to stereotactic frame fixation. High blood pressure (systolic pressure > 130–150 mmHg) values were treated with calcium blockers.

Awake group: Patients were given only local anesthetics prior to skin incision. No other anesthetics were given during the procedure.

Ketamine group: Patients followed a propofol–ketamine–propofol sedation paradigm (Baker Erdman et al. 2022; Kornilov et al. 2024) where propofol was administered starting after the patient was positioned on the operating room table and was stopped ~15 min prior to MER initiation. Ketamine infusion then began. Typical drip rate of ketamine was between 0.25 and 0.1 mg/kg/h, resulting in a moderate sedation level as defined by the American Society of Anesthesiologists. Median total ketamine dose for this group was 46.5 mg (the typical duration of one MER trajectory equals 30 min).

N_2_O group: Patients were deeply anesthetized, paralyzed, intubated and ventilated, and maintained under IV propofol before and during frame fixation and CT acquisition. Upon their return to the OR, propofol was stopped, and they were administered 60%–70% N_2_O. Ketamine was added if needed to keep patients deeply anesthetized. The typical drip rate of ketamine was between 0.25 and 0.5 mg/kg/h. Average total ketamine based on available data for this subgroup (23/37 patients) was 88.4 mg/procedure (2–3 h).

### 2.6 Electrophysiological Data Collection

One or two microelectrodes were placed 10 mm above the planned target (5 mm below the lower end of the cannula), and recordings were made in steps of 0.4 mm prior to STN entrance and in steps of 0.1 mm within the STN using the NeuroOmega navigation system (Alpha Omega Engineering, Nof HaGalil, Israel). Recording at each site began after a 2‐s stabilization period and lasted 4 s, for a total of 6 s at each site. STN entrance was identified as an increase in background noise from baseline levels, high density firing, and usually an increase in theta and beta frequency bands. In some cases, STN entry revealed an increase in the power of the delta frequency band, as visualized using the real‐time analysis and automatic algorithm of the HaGuide (Alpha Omega Engineering, Nof HaGalil, Israel). Though beta oscillatory activity can be found in surrounding nuclei, there is generally a clear increase in the beta frequency band upon entering the STN from the internal capsule or thalamus along the typical DBS trajectory. If an increase in the power of the beta and lower frequency bands was noted, this was used as a marker for the motor domain of the STN. The ventromedial region (non‐motor domain) of the STN was designated by broad frequency gamma activity and spanned the remaining part of the trajectory. STN exit was set as the point in which background noise level decreased and high density firing ended or electrophysiological signs of the substantia nigra reticulata were observed (Valsky et al. 2017).

### 2.7 Electrophysiological Data Analysis

Electrophysiological data analysis was identical to that reported in Baker Erdman et al. (2022). Namely, spiking activity (300–6000 Hz band‐pass filtered) was extracted from the NeuroOmega navigation system (Alpha Omega Engineering, Nof HaGalil, Israel). Power spectrum density (PSD) and root mean square (RMS) were computed for each recording site. The RMS was normalized to the RMS level of the first 5–6 sites taken 0.1 mm apart, 10 mm above the planned target while still outside the STN, and usually in the internal capsule. The PSD is calculated from the rectified (absolute operator) data and normalized to the RMS (total power in the recorded frequency range). Thus, the PSD gives detailed information about the distribution of power of the envelope of the spiking activity at discrete frequency bins (1/3 Hz resolution) as a fraction of the total power at a site (i.e., it is a measure that is not affected by changes in the total power). STN borders and subdomains were determined using a previously published algorithm (Valsky et al. 2017; Zaidel et al. 2009) and corrected (if needed) by an expert electrophysiologist (H.B.E.).

If two electrodes were used, only the trajectory that was chosen for implantation was included in this study. The recorded STN length was extracted from trajectory recordings for each patient, and the length was normalized to a STN length of 1 to enable group analysis. For each trajectory, a segment before STN entrance that is equal to half of the length of the STN from that trajectory is included in our normalized visualizations. PSD and normalized RMS (NRMS) for the STN of individual patients were then grouped and averaged to create an average PSD and NRMS for each patient group.

Beta band data were extracted using a four‐pole band‐pass filter from 13 to 30 Hz. Due to the lack of increase in the power of the beta band in the individual trajectories of the N_2_O group, the percent motor subregion was analyzed using the delta range on the averaged PSD and not on individual trajectories. The amount of beta power within the motor subregion (taken as the first 1/4 of the STN due to reduced ability to distinguish the motor region in individual trajectories of the N_2_O group) compared to the amount of beta power outside the STN was calculated as the beta ratio for each trajectory and then analyzed across groups. Using the first half of the STN to calculate the motor subregion yielded similar results. The peak value and area under the curve (AUC) of the normalized RMS was computed for each trajectory and then evaluated across groups. All analysis was performed using custom Matlab (v2017a) scripts.

### 2.8 Single Unit Analysis

For each patient, we detected the STN borders and depth using the NeuroOmega navigation system and automatic algorithm of the HaGuide (Alpha Omega Engineering, Nof HaGalil, Israel). For each file, the neural data was bandpass filtered (300–3000 Hz) to isolate spike signals. Spike detection was performed by identifying peaks in the filtered signal that crossed a threshold, defined as four times the standard deviation of the noise. A refractory period of 1.5 ms was applied to ensure the physiological validity of spike events. If a spike was detected, we checked for additional spikes within a window of 1 ms before and 2 ms after the detected spike. If another spike was found within this interval, it was excluded. Firing rates were determined as the total number of spikes divided by the recording duration. Coefficient of variation of interspike interval (CV‐ISI), a measure of firing variability, was computed as the standard deviation of interspike intervals (ISI) divided by their mean. The ISIs was calculated for each patient, and the mean and standard deviation of all ISIs were used to compute the CV‐ISI. Mean firing rates and their CV‐ISI were similarly calculated for each patient and then averaged. There was no robust difference between the results of the two methods. Finally, group‐level averages of these metrics were computed across 10 patients per experimental group to enable comparative analysis. Outliers were removed from the analysis using interquartile range thresholds to ensure robust statistical representation.

We calculated the spike‐triggered average (STA) of LFP signals from neural recordings. For each detected spike, a window of 1 s before to 2 s after the spike time was extracted from the LFP data recorded by the same microelectrode. These spike‐centered LFP windows were collected across all files into a cumulative STA matrix. After processing all files, the STA was averaged across all spike‐centered windows. A similar process was repeated to detect LFP beta oscillations except that a bandpass filter of 13–30 Hz (fourth‐order non‐causal Butterworth filter) was used in place of the LFP filter.

### 2.9 Statistical Data Analysis

After the preparation of the database, statistical assumptions were tested. In the case of normal distribution (electrophysiological data parameters), parametric tests were used. Nonparametric tests were used for non‐normally distributed data (demographic patient data). Results are presented as mean ± standard error mean (SEM) for continuous normally distributed variables, average ± standard deviation for non‐normally distributed data, and number (%) for categorical variables. Analysis of variance (ANOVA) or Kruskal–Wallis test were used for group comparisons. Significance was determined using a *p*< 0.05.

## 3 Results

### 3.1 STN Gross Electrophysiological Dimensions Do Not Differ Between Groups

**FIGURE 1:**
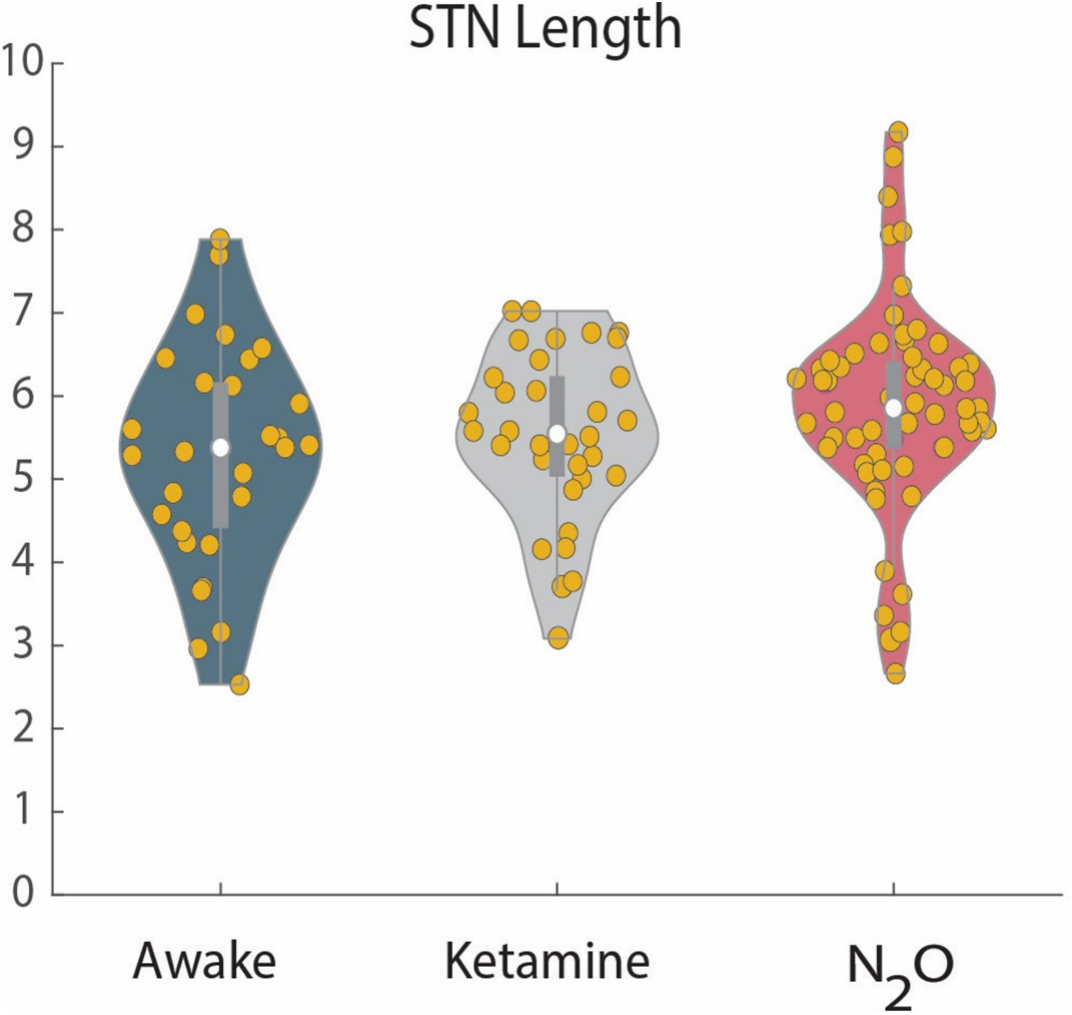
Total STN electrophysiological length does not differ between groups. Blue = awake, grey = ketamine, red = nitrous oxide. Yellow dots represent individual trajectories. White dot represents the median, dark grey bar represents the 25th and 75th percentile, and top and bottom grey lines illustrate the maximum and minimum for each group.

Total STN electrophysiological length along the trajectories in the three groups ranged from 3 to 8 mm, with an average total STN length of 5–6 mm. Total electrophysiological STN length was not significantly different between groups (Figure 1; *p* = 0.07). Motor subregion length and ventromedial region length could not be reliably delineated in individual trajectories in the N_2_O–ketamine group and therefore could not be compared with the other two groups. However, when normalized and averaged, some increase in the beta frequency, and a noticeable difference in the delta frequency range, can be observed in the N_2_O group, probably representing the motor subregion, whereas broad gamma oscillations signify the ventromedial (non‐motor) subregion. The percentage of the total STN length that is designated as motor subregion in the averaged N_2_O group is 64.6% compared to 57.9% and 56.1% in the awake and ketamine groups, respectively.

### 3.2 N_2_O Decreases the Overall Activity Level Inside the STN

**FIGURE 2:**
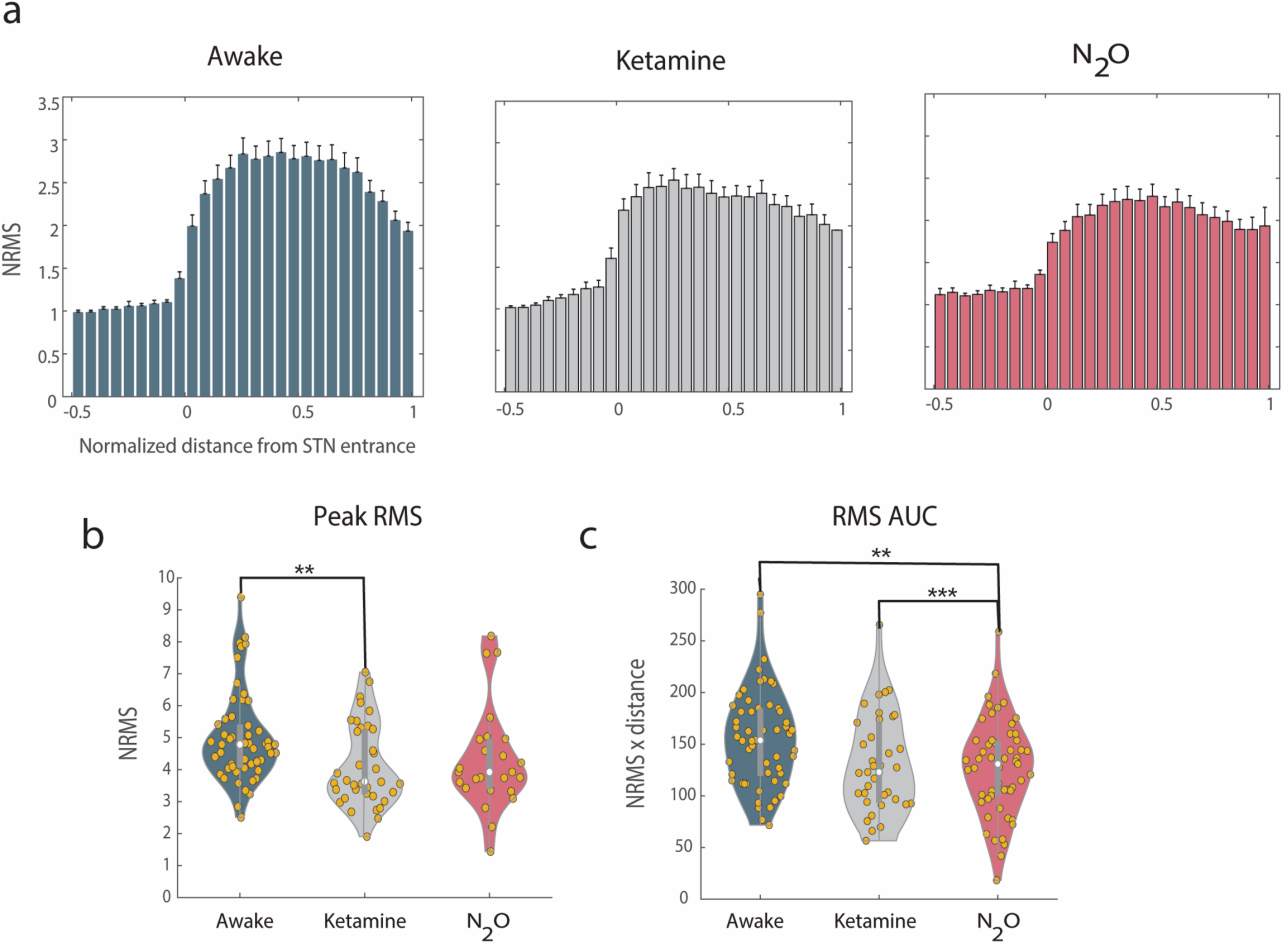
RMS significantly differs between groups. (a) The group averaged normalized RMS for each group as a function of normalized distance from STN entrance. (b) Peak NRMS values for each group. (c) The area under the curve of the NRMS for each group. ANOVA revealed significant differences in peak and AUC NRMS; post hoc *t*‐test results (Bonferroni corrected) are denoted by asterisks. Coloring and violin plots are in the same convention as Figure 1. Yellow dots represent individual trajectories. Error bars on bar graphs illustrate standard error mean.

RMS analysis revealed that the awake group shows a clear and substantial increase in the normalized RMS (NRMS) at the entrance of the STN that continues to its exit (Figure 2a). The N_2_O group's RMS increase was smaller, though clear. In contrast to our previous findings (Baker Erdman et al. 2022), the ketamine group also showed some attenuation but to a lesser extent than the N_2_O group. To quantify these differences, we calculated the peak value of the NRMS and the AUC of the NRMS. The peak value of the NRMS significantly differs between groups (*p* = 0.0085; Figure 2b). Likewise, the AUC of the NRMS is significantly different between the three groups (*p* = 0.003; Figure 2c). As RMS can be thought of as a measurement of general activity level in the area surrounding the electrode, we suggest that the overall activity level in the STN is decreased under N_2_O–ketamine general anesthesia compared to awake.

### 3.3 N_2_O Significantly Attenuates Beta Frequency Power Inside the STN

**FIGURE 3:**
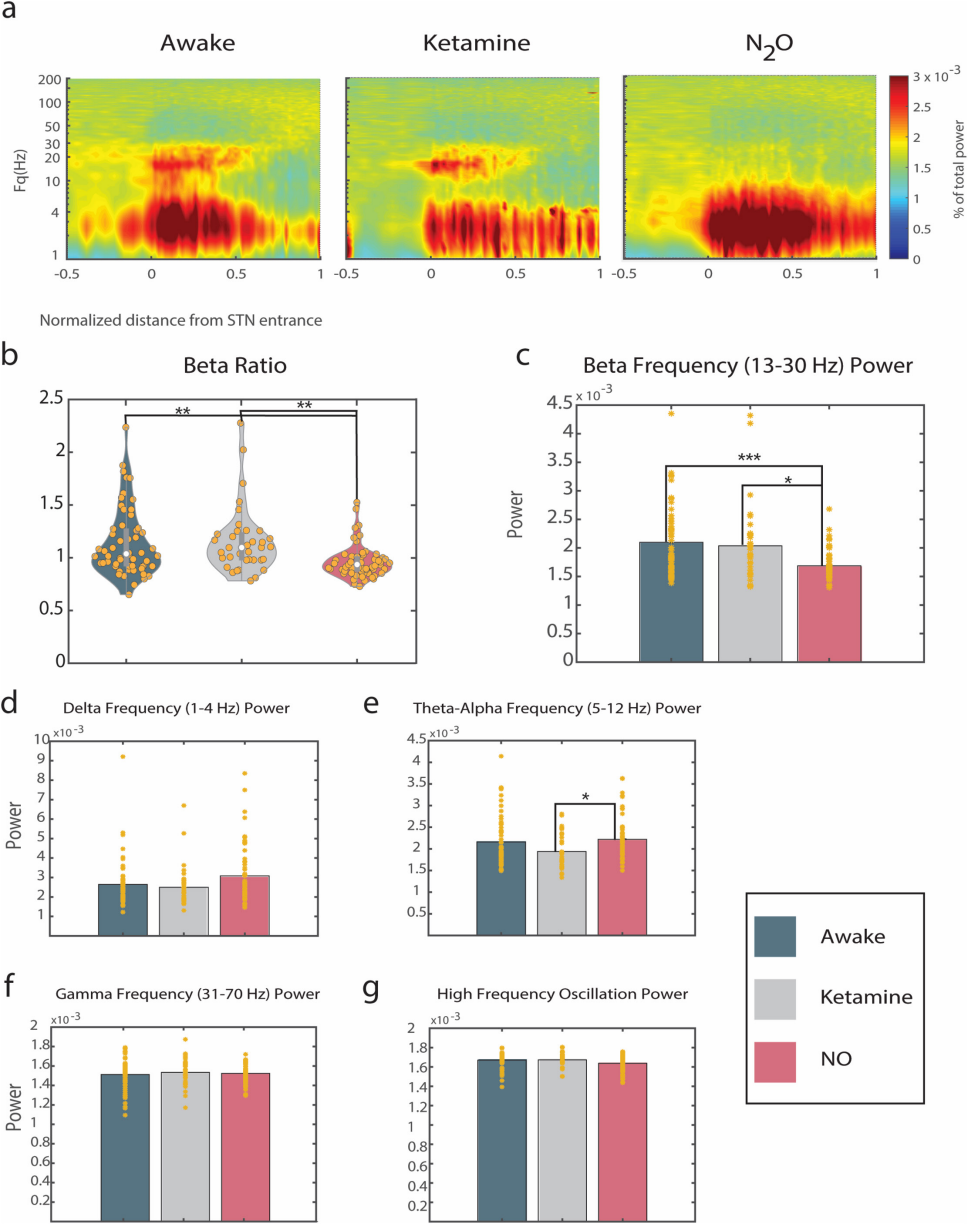
N_2_O significantly attenuates beta frequency power in the STN. (a) Group averaged spectrograms. The heat map represents the percent of total power. On the x‐axis, −0.5 indicates half the length of the STN prior to STN entrance, 0 indicates entrance to STN, and 1 indicates exit from STN. (b) The beta frequency power ratio (beta frequency power inside motor subregion STN: beta frequency power before STN entrance) for each group using the same conventions as previous violin graphs. (c–g) Power for respective frequency bands as a group (bar level) and individual trajectories (yellow stars). Post hoc *t*‐test results (Bonferroni corrected) are denoted by asterisks.

To investigate changes in the frequency distribution across groups, spectral analysis was performed. Figure 3a shows the averaged spectrogram for each group calculated with a frequency range between 1 and 200 Hz. In the awake and ketamine groups, the entrance to the motor subregion of the STN (marked 0) is clearly identified by an increase in power in the beta frequency range (13–30 Hz). This signature is greatly attenuated in the spectrogram of the N_2_O group.

**FIGURE 4:**
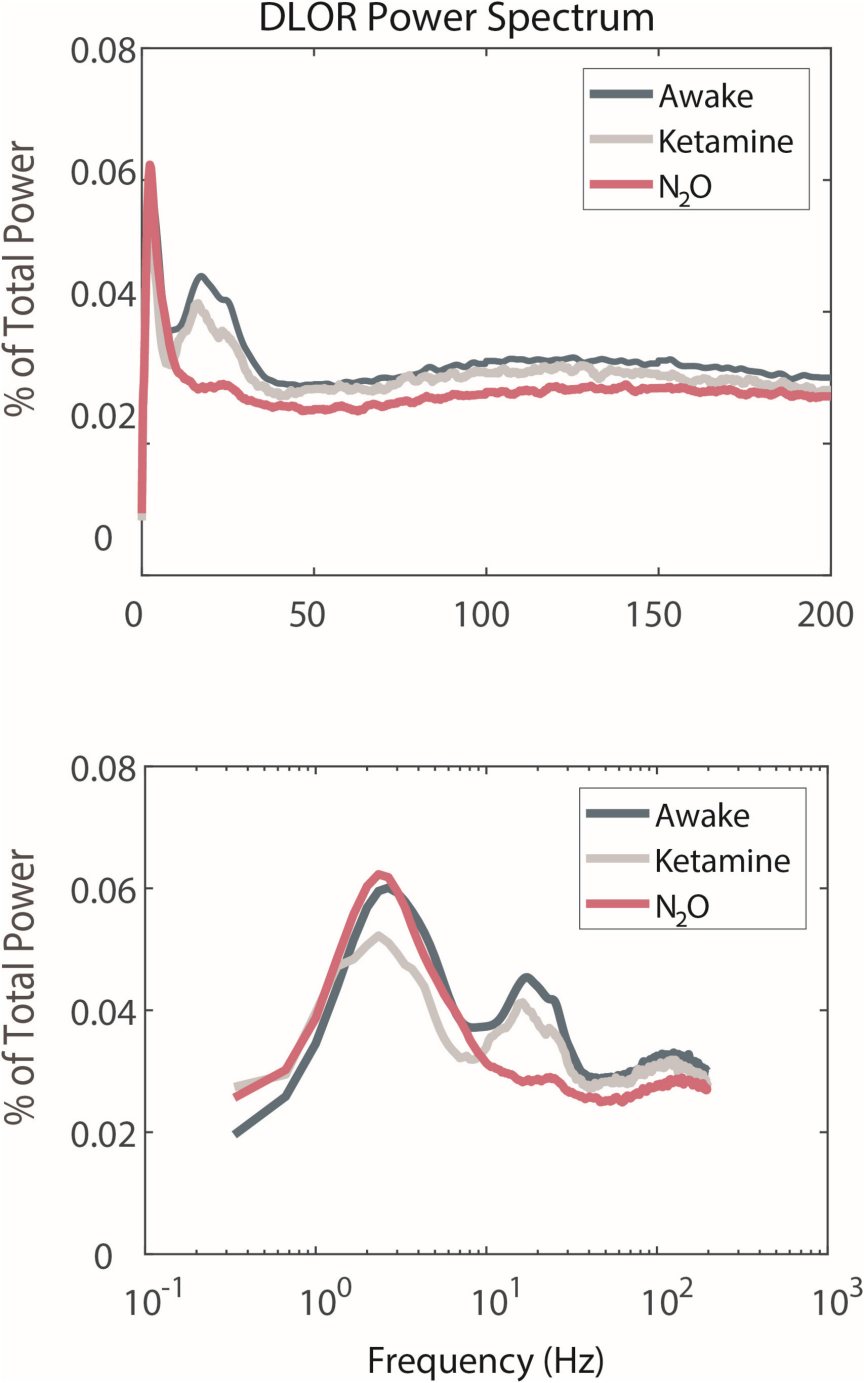
Beta frequency differences in the motor subregion of the STN. Top graph shows the power spectrum within the motor subregion on standard scale. Bottom graph shows the same data in log scale. Coloring as in previous figures.

The beta ratio, that is, the beta frequency power inside the motor subregion of the STN in relation to the beta frequency power prior to STN entrance, significantly differed between groups (*p* = 0.0003; Figure 3b). Similarly, the mean power of beta frequency domain (13–30 Hz) inside the motor subregion was found to be significantly different (*p* < 0.0001; Figure 3c). Power distribution changes were also observed in the delta frequency band power (1–4 Hz, *p* = 0.048; Figure 3d), the theta‐alpha frequency band power (4–12 Hz, *p* = 0.03; Figure 3e), and in the high frequency (70–150 Hz) range (*p* = 0.04; Figure 3g) but not in the gamma frequency band power (31–70 Hz, *p* = 0.68; Figure 3f). A closer inspection of the differences, specifically within the motor subregion of the STN, shows a peak in the beta frequency range at ~17 Hz in the awake and ketamine groups but no peak in this range in the N_2_O group (Figure 4a,b). Though it is not present in all individual trajectories, there is an increase in the delta frequency band power in the average spectrogram for this group at ~3 Hz.

### 3.4 STN Firing Rate Decreases Under N_2_O

**FIGURE 5:**
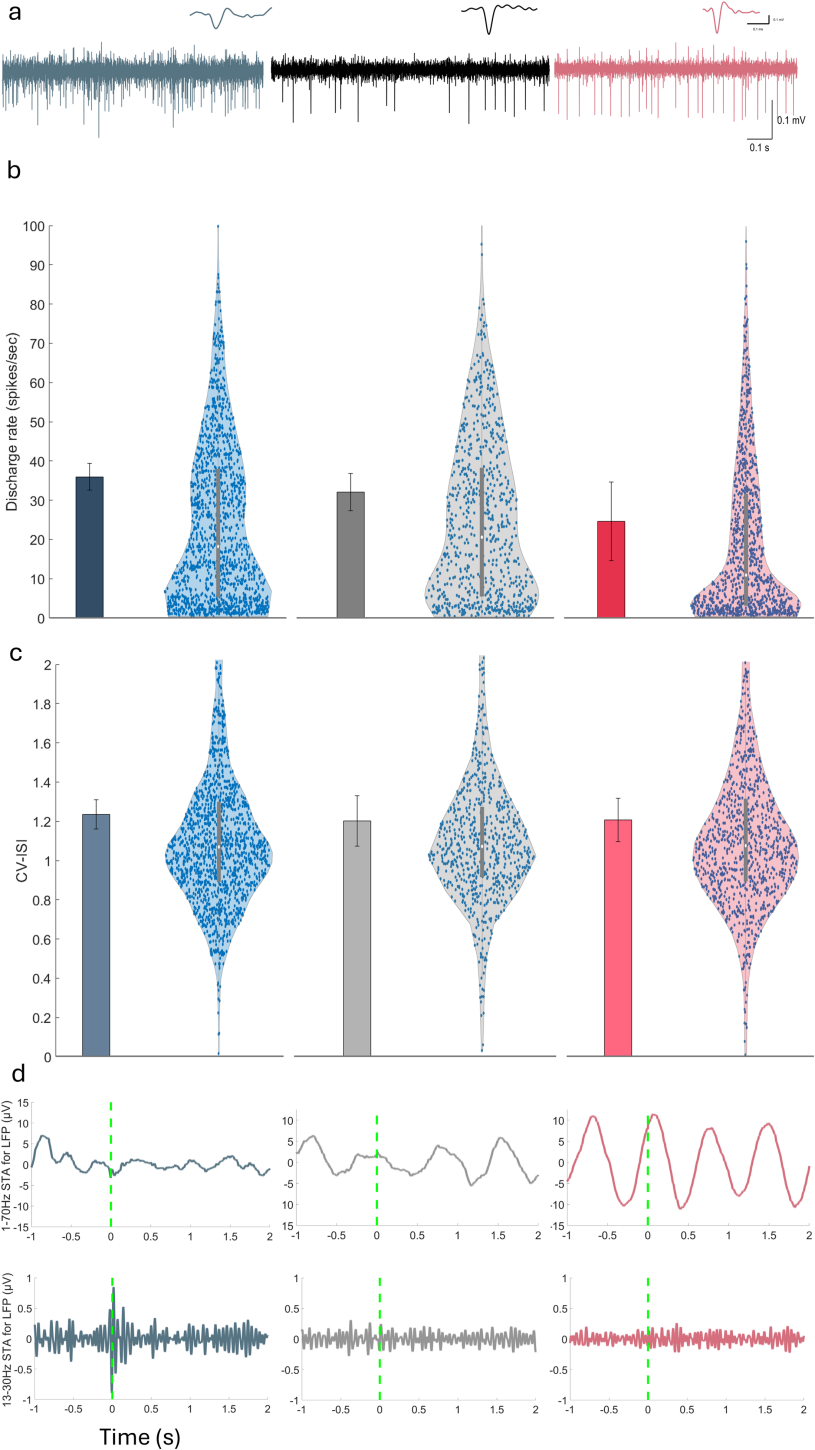
Spiking activity modulation in the STN. (a) Representative raw neural activity traces for each experimental group. A 1‐s segment of the neural signal (amplitude in microvolts, μV) is plotted against time in seconds. (b) Bar plots showing the mean firing rate (spikes/s) and the coefficient of variation of interspike intervals (CV‐ISI) for the three experimental groups along with the standard variation. (c) Violin plots display the distributions of firing rates (left) and CV‐ISI values (right) for the three experimental groups. (d) Local field potential (LFP) analysis and spike‐triggered average (STA) plot. The raw neural signals were filtered using a 1–70 Hz bandpass filter to detect the LFP (top) and 13–30 Hz bandpass filter to show the beta oscillations (bottom). Coloring as in previous figures.

The average firing rate differed significantly (*p* = 0.0028) between the three groups. It was 35.95 (+/−3.42) spikes/s in the awake group, 32.08 (+/−4.79) spikes/s in the ketamine group, and 24.57 (+/−10.04) spikes/s in the N_2_O group (Figure 5a,b). Post hoc testing revealed that the N_2_O group differed significantly from both the ketamine (*p* = 0.049) and awake group (*p* = 0.0023). The ketamine and awake groups did not differ (*p* = 0.42). The firing pattern, as measured by CV‐ISI, was not significantly different between groups (*p* = 0.76; Figure 5d). However, a deeper look at the spiking activity using the spike‐triggered average (STA) exposed significant entrainment of spiking activity in the STN to LFP delta waves. This entrainment was much more robust for the N_2_O group. On the other hand, beta entrainment was stronger for the awake and the ketamine groups (Figure 5d).

## 4 Discussion

This study aimed to characterize electrophysiological changes that occur in the STN of PD patients undergoing DBS procedures in response to N_2_O general anesthesia supplemented by ketamine compared to patients under low‐dose ketamine sedation or awake states. Our data suggest that the overall activity level of the STN is decreased by a small but statistically significant amount during N_2_O–ketamine general anesthesia compared to awake but is similar to that under low‐dose ketamine preceded by propofol. Further, the power of the beta frequency band is drastically mitigated compared to both the low‐dose ketamine and awake groups. Firing rate, but not pattern, is also diminished in the N_2_O–ketamine group. Practically, this makes the delineation between the motor and non‐motor subregions of the STN difficult. However, it is still possible to recognize with high confidence the entrance and exit of the nucleus. Finally, when we investigated the spiking activity, the STA‐LFP showed that there was significant entrainment by the LFP delta oscillations. This finding suggests that the STN delta LFP is largely generated in the STN and/or reflects volume conductance of major STN afferent activity such as the cortex, thalamus, or hypothalamus.

DBS surgery is becoming increasingly common due to surgical improvements, the growing population of PD, and the expansion of DBS as a treatment for other diseases. Along with the GPi, the STN remains the most common target for DBS surgery. Although this can be done using only imaging techniques (Foltynie et al. 2011; Pollo et al. 2003; van den Munckhof et al. 2021), MER‐guided DBS provides an extra failsafe to make up for brain shift, distorted imagining, inaccurate coregistration of images, and human errors. Additionally, MER gives a higher spatial resolution of the nucleus and enables delineation of its motor and non‐motor subdomains.

Aberrant beta frequency oscillations in the basal ganglia are well documented in PD (Bergman et al. 1994; Brown et al. 2001; Levy et al. 2000, 2002; Miller and DeLong 1987). Attenuation of the beta band power and coherence in the STN have been shown to occur as a result of dopamine replacement therapy (Levy et al. 2001; Priori et al. 2002) and DBS surgery (Quinn et al. 2015), suggesting that this is a pathological feature of the disease. Moreover, severity of PD motor symptoms has been correlated with the degree of beta activity in the STN (Asch et al. 2020). Hence, the beta frequency power is an important marker of the STN when performing DBS surgery with MER. Due to its uniqueness along the typical DBS trajectory, an increase in beta power is a reliable sign that we have entered the STN and not another high‐density firing area such as the thalamus or red nucleus. The end of the beta power increase signals the transition from the motor subregion to the non‐motor subregion of the STN (Shamir et al. 2013; Zaidel et al. 2010). Properly recognizing this border allows for optimal placement of the lead and enhances lead programming efficacy and flexibility. Relatedly, accurate beta activity power measurement during the trajectory allows for easier identification of ideal stimulation parameters postoperatively due to the correlation between stimulation of the lead contact with the highest beta and symptom improvement (Chen et al. 2020). As such, disruption of the beta band in the STN by N_2_O–ketamine general anesthesia is a significant disadvantage and should be taken into consideration when choosing the anesthetic protocol for DBS surgery. Beyond this disadvantage, there are other drawbacks and benefits to consider when using N_2_O general anesthesia for DBS surgery. The main disadvantage with any general anesthetic, including N_2_O, for DBS surgery is the inability to perform intra‐operative stimulation testing. In awake or ketamine‐sedated patients, the stimulation can be delivered using the macro‐contact of the MER electrode before being replaced with the permanent lead. In our practice, this intraoperative stimulation testing occurs after the target nucleus has been identified usually with the macro‐contact positioned just above the motor/non‐motor subregion border. The stimulation testing allows the surgeon to assess the therapeutic window by evaluating at what amplitude (frequency and pulse width are usually kept constant at typical DBS settings) symptoms like rigidity, bradykinesia or tremor start to improve and at what point side effects such as gaze palsy, dysarthria and/or orofacial contraction, or temporary paresthesia appear. A low threshold for side effects can be indicative of slight or severe misplacement and can be of great help in adjusting position prior to lead implantation. In some centers, kinesthetic testing of the patient's limbs during the MER phase is used to identify the motor subregion of the STN. Due to the lack of clear beta frequency signature, this technique could have been useful for patients undergoing DBS under general anesthesia. However, muscle relaxants are often used in fully anesthetized patients, as was the case in our cohort, making kinesthetic testing impossible. Evoke resonant neural activity (ERNA) may compensate for this issue (Wiest et al. 2023). Additionally, there is a concern for increased brain shift because of N_2_O general anesthesia due to pneumocephalus expansion. There are reports suggesting N_2_O causes pneumocephalus expansion due to its ability to diffuse into a closed gas space significantly faster than air, increasing the volume of the space, potentially causing brain shift and increasing intracranial pressure (Artru 1982; Mallamo et al. 1975; Pandit et al. 1982; Raggio et al. 1979; Saidman and Eger 1965; Singh et al. 2015; Skahen et al. 1986). This could manifest in a higher number of electrode trajectories if MER is employed or an increase in mis‐implantations if direct targeting is used. However, other studies reported no such danger when using N_2_O general anesthesia (Di Lorenzo et al. 1986; Friedman et al. 1981; Moseley et al. 1977). As such, further investigation is necessary to determine with confidence whether this should be a concern.

On the other hand, there are major advantages of N_2_O general anesthesia as well. Awake neurosurgery comes with many challenges such as patient stress and agitation that, if severe, can result in aborted surgery. With the patient fully anesthetized, premature surgery termination, at least for this reason, is not a concern. The well‐being and experience of the patient during surgery is also likely to be significantly improved compared to the surgery being performed awake. Additionally, there are patients in which general anesthesia is needed (e.g., very young children operated on for genetic dystonia). In these cases, N_2_O augmented by ketamine, at least during the physiological navigation phase, is preferable to hypnotic anesthesia or propofol TIVA because the target nucleus can still be identified. Some centers choose to use dexmedetomidine during DBS surgeries and claim good electrophysiological results, at least when used in low doses for sedation (Martinez‐Simon et al. 2017) or when added to a cocktail to induce anesthesia (Mathews et al. 2017). Yet, the firing rate of neurons in the pallidum was shown to decrease in a dose‐dependent manner (Gasim et al. 2021), so it is unclear how reliable general anesthesia maintained by dexmedetomidine alone would be in identifying the closely connected STN. Other centers rely on remifentanil‐based anesthesia and similarly claim satisfactory MER results (Vesper et al. 2022). A direct comparison between nitrous oxide, dexmedetomidine, and remifentanil general anesthesia MER is certainly warranted to determine the optimal choice for DBS surgery.

## 5 Conclusions

In summary, N_2_O–ketamine general anesthesia allows for satisfactory MER localization of the STN despite a decrease in overall activity level. However, its use could obscure the motor/non‐motor border due to decreases in the beta frequency power signature, which is valuable for the lead placement decision. This may affect DBS outcome due to limitation of programming abilities and increase surgical revision rates for suboptimally placed leads. However, the current study did not investigate these postoperative effects and clinical outcome.

The small sample size and retrospective nature restrict the conclusions of this study. Likewise, the lack of a control group under a different type of general anesthesia limits our findings as we cannot conclusively determine if the changes we observed were due to depth of anesthesia or N_2_O specifically. Entropy values were tested in several patients in the N_2_O group and showed somewhat steady values above 80 with sudden, unexplained dips to almost zero. This aligns with previous studies demonstrating that frontal EEG measurement of the depth of anesthesia (bispectral index, BIS, included) may not be reliable under nitrous oxide (Foster and Liley 2011). Additionally, there was no patient matching by demographics or clinical symptoms across groups that could have a significant effect on the results. Patients were also gathered from different medical centers, so there were differences in presurgical planning and variations in surgical procedure. However, the electrophysiology team was consistent for all surgeries. We therefore believe that, considering the increasing number of DBS surgeries being performed worldwide and the desire to improve patient and surgeon experience, the use of N_2_O anesthesia in DBS procedures is valuable for selected cases and centers and justifies future research.

## Author Contributions

**Halen Baker Erdman:** conceptualization, data curation, formal analysis, investigation, methodology, resources, writing – original draft, writing – review and editing. **Hagai Bergman:** conceptualization, data curation, funding acquisition, methodology, project administration, resources, writing – original draft, writing – review and editing. **Karin Abu Haya:** formal analysis, writing – review and editing. **Stefanie Glowinsky:** formal analysis, writing – review and editing. **Lotem Warhaftig:** data curation. **Juan F. León:** data curation, resources, writing – review and editing. **Zvi Israel:** data curation, investigation, writing – review and editing. **Muneer Abu Snineh:** data curation, formal analysis. **Evgeniya Kornilov:** data curation, formal analysis, writing – review and editing. **Omer Zarchi:** data curation, writing – review and editing. **Idit Tamir:** data curation, investigation, validation, writing – review and editing. **Johnathan Reiner:** data curation, writing – review and editing. **Tsvia Fay‐Karmon:** data curation, resources, writing – review and editing. **Sharon Hassin‐Baer:** data curation, writing – review and editing. **Violeta Glauber:** data curation, investigation, writing – review and editing. **Tomer Nir:** conceptualization, data curation, investigation, writing – review and editing. **José Antonio Asprilla González:** data curation, investigation, writing – review and editing. **Lior Ungar:** data curation, investigation, writing – review and editing. **Zion Zibly:** conceptualization, data curation, investigation, methodology, project administration, resources, writing – original draft, writing – review and editing.

## Disclosure

Halen Baker Erdman is an employee and holds a patent with Alpha Omega, Israel. Hagai Bergman is a consultant who has several patents with Alpha Omega, Israel. He received travel honoraria for lectures, presentations, and speaker's bureaus from Medtronic, Boston Scientific, and Alpha‐Omega.

## Conflicts of Interest

The authors declare no conflicts of interest.

### Peer Review

The peer review history for this article is available at https://www.webofscience.com/api/gateway/wos/peer‐review/10.1111/ejn.70039.

## Data Availability

Anonymized data can be provided on request from the corresponding author. It has not been uploaded to a public database for confidentiality reasons.
